# Management of Pleural Effusion Secondary to Malignant Mesothelioma

**DOI:** 10.3390/jcm10184247

**Published:** 2021-09-19

**Authors:** Valeria Musso, Cristina Diotti, Alessandro Palleschi, Davide Tosi, Alberto Aiolfi, Paolo Mendogni

**Affiliations:** 1 Thoracic Surgery and Lung Transplantation Unit, Fondazione IRCCS Ca’ Granda Ospedale Maggiore Policlinico, 20122 Milan, Italy; valeria.musso@unimi.it (V.M.); cristina.diotti@unimi.it (C.D.); alessandro.palleschi@unimi.it (A.P.); davide.tosi@policlinico.mi.it (D.T.); 2 Department of Pathophysiology and Transplantation, University of Milan, 20122 Milan, Italy; 3 Department of Biomedical Science for Health, University of Milan, 20133 Milan, Italy; alberto.aiolfi86@gmail.com; 4 Division of General Surgery, Istituto Clinico Sant’Ambrogio, 20149 Milan, Italy

**Keywords:** malignant pleural effusion, malignant pleural mesothelioma, mesothelioma, indwelling pleural catheter, talc poudrage

## Abstract

Malignant pleural mesothelioma (MPM) is a highly aggressive pleural tumour which has been epidemiologically linked to occupational exposure to asbestos. MPM is often associated with pleural effusion, which is a common cause of morbidity and whose management remains a clinical challenge. In this review, we analysed the literature regarding the diagnosis and therapeutic options of pleural effusion secondary to mesothelioma. Our aim was to provide a comprehensive view on this subject, and a new algorithm was proposed as a practical aid to clinicians dealing with patients suffering from pleural effusion.

## 1. Introduction

Malignant pleural mesothelioma (MPM) is a highly aggressive tumour of the pleura, which has been epidemiologically linked to occupational exposure to asbestos [1], whose incidence is still growing due to the long latency period of the disease [2]. Clinical presentation of patients suffering from MPM is characterized by a variable grade of dyspnoea, cough and chest pain, related to chest wall invasion, to the onset of pleural effusion, or to a combination of both. Specifically, the effusion is caused by the imbalance between the amount of pleural fluid produced and its resorption due to the increased vascular permeability and inadequacy of the lymphatic drainage system, respectively. The volume of pleural effusion, together with the severity of pain, as well as patient’s cardiopulmonary function, determines the grade of dyspnoea.

Pleural effusion remains a common clinical condition, affecting more than 1.5 million patients per year in the USA alone [3]. In general, the approach to the patient with pleural effusion is challenging and is outlined as a multi-step clinical path: from the assessment of nature and origin of the effusion to the management of pleural space, to the treatment of the underlying disease. The differential diagnosis is mostly based on patients’ clinical history, diagnostic imaging, and pleural fluid analysis or pleural biopsy.

In this review, we will focus on malignant pleural effusion secondary to pleural mesothelioma: we analysed the literature on the diagnosis and therapeutic options of pleural effusion secondary to MPM. Our aim was to provide a comprehensive view on this interesting topic; then, we try to draw a new and comprehensive algorithm for the diagnosis and management of these patients.

## 2. Materials and Methods

We searched PUBMED using the following search string: (“Mesothelioma” [Mesh: NoExp]) AND (“Pleural Effusion, Malignant/drug therapy” [Mesh] OR “Pleural Effusion, Malignant/prevention and control” [Mesh] OR “Pleural Effusion, Malignant/radiotherapy” [Mesh] OR “Pleural Effusion, Malignant/surgery” [Mesh] OR “Pleural Effusion, Malignant/therapy” [Mesh]).

Articles in languages other than English and case reports were excluded. All articles retrieved were then selected by two authors (CD, VM) based on title and abstract; the final selection was carried out after reading the full text.

## 3. Pathophysiology and Aetiology of Pleural Effusion

Pleural effusion has a wide variety of aetiologies, which can be classified according to the nature of the pleural fluid: transudative (usually related to an imbalance in hydrostatic or oncotic pressure) or exudative (generally caused by an increase in vascular permeability, inflammation or lymphatic drainage obstruction). Pleural fluid is classified as transudate or exudate based on the Light’s criteria. Pleural effusion can be further categorized as benign or malignant. Malignant pleural effusion is the second most common cause of exudative pleural effusion after parapneumonic effusion [2]. It accounts for about 15–35% of pleural effusions in general. The most common neoplasms determining pleural effusion are advanced lung, breast, gynaecological, gastrointestinal and haematological neoplasms or primary pleural tumour (i.e., MPM); however, every tumour with pleural metastasic spread can potentially cause pleural effusion. The prevalence of pleural effusion in MPM is high, ranging from 54 to 90% [4]. The main mechanisms underlying pleural effusion, both from benign and malignant causes, are the increase in capillary pressure and permeability of the pleura, and a reduced flow in draining lymphatic vessels. In case of malignant pleural effusion, the causes are several: the direct obstruction of lymphatics and pleural capillaries by the tumour or pathologic nodes; the involvement of the pericardium, a superior vena cava syndrome or right heart compression and tamponade can also be responsible for pleural effusion. Moreover, neoplasms can produce vasoactive substances, such as vascular endothelial growth factor (VEGF), which determine an inflammatory response and an increase in vascular permeability. Finally, systemic effects related to the tumour, such as cancer-related hypoalbuminemia and the hypercoagulable state cause a reduced oncotic pressure and pulmonary embolism respectively, which can increase pleural fluid accumulation.

## 4. Diagnosis

Pleural effusion usually occurs early in the mesothelioma course, and it is, therefore, responsible for the presenting symptoms. The most common symptoms are a progressively worsening dyspnoea, weight loss, fatigue, chest pain and discomfort, and cough. The severity of the dyspnoea is associated both with the amount of pleural fluid and with the rate of fluid accumulation, but it is also affected by underlying cardiologic and respiratory comorbidities.

Imaging has a relevant role in determining MPM as the cause of pleural effusion, mainly because it allows for the detection of those coexisting findings which are suggestive for malignancy. Pleural effusion is often demonstrated for the first time by performing a chest X-ray: a 200 mL pleural effusion can be detected on chest X-ray in the posterior-anterior view. A massive pleural effusion is frequently related to malignancy. In case of effusion with no history of cardiopathy and a normal heart size on chest X-ray, cancer should be considered as a likely diagnosis, even though systemic lupus and pleural effusion due to liver failure can have a similar clinical presentation. Except for direct invasion by lung or breast cancer, which are frequently associated with ipsilateral pleural effusion, bilateral effusion is more common in case of other metastatic primary tumours [5]. In malignant mesothelioma, pleural effusion is usually unilateral, and common chest X-ray findings are pleural plaques and nodules. Notably, even in patients with a large pleural effusion, contralateral mediastinal shift is often absent due to the rigid thickened pleural tissue encasing the ipsilateral lung. Chest ultrasound (US) can detect pleural effusion and is more sensitive than chest X-ray, with a 70–80% sensitivity and 84–100% specificity [6]. This technique allows to assess possible pleural thickening and pleural or diaphragmatic nodules, which can be suggestive of malignancy. Moreover, septations or loculated pleural effusion can be detected using US: this can occur in case of malignant pleural effusion, but can also indicate an inflammatory process. Since loculations can also prevent a complete drainage of the pleural effusion and therefore lung re-expansion, their presence should be taken in consideration when managing loculated pleural effusion. US provides both real-time and dynamic information and it is non-invasive, portable and simple to perform, thus representing an ideal tool particularly when pleural effusion monitoring is required. Moreover, ultrasound is used to guide invasive procedures such as thoracenteses, to assess lung re-expansion, and the presence of pneumothorax. Computed tomography (CT) with intravenous contrast is the most informative radiological technique when assessing pleural effusions, and it is mandatory in order to evaluate the extent of the disease and determine the best diagnostic procedure. Nodular or diffuse pleural thickening, particularly when associated with involvement of the mediastinal pleura, is indicative of malignant pleural mesothelioma. CT scan alone does not provide a definitive diagnosis [7]. Pleural plaques are often found on CT scans of patients with a previous exposure to asbestos, which makes it challenging to determine whether they could be an independent factor associated with malignant pleural mesothelioma [8]. The presence of enlarged lymph nodes or other lesions consistent with primary cancer can guide the diagnostic process and provide information for disease staging. CT scan can also help discriminate between benign pleural lesions and malignancy; a CT-scan-based scoring system has been proposed to predict malignancy [9]. In case of pleural effusion, CT scan should preferably be performed after its evacuation, as this allows to evaluate the lungs and the other structures without the disturbance caused by the presence of the fluid. Positron emission tomography (PET) scan with fluorodeoxyglucose is used to obtain functional data on pleural lesions, to complete the staging, and to target the most suitable area in case biopsies are required. The positive predictive value is reduced in patients after pleurodesis, particularly when performed with talc, or in case of overlapping infections [5,10]. The role of magnetic resonance imaging (MRI) in the diagnosis of malignant pleural mesothelioma is yet to be established and validated [7]; this technique could provide data on chest-wall and diaphragmatic involvement but is less informative with regard to the lung tissue; also, many centres have limited access to MRI. Thoracentesis has both a diagnostic and therapeutic aim in suspected malignant pleural effusion. The procedure must be performed with aseptic technique, after local anaesthesia. The use of US guidance has contributed to reducing the most common complications after thoracentesis and is recommended by the guidelines drafted by the major scientific societies [7]. In patients with symptomatic malignant pleural effusion, large-volume thoracentesis (i.e., drainage of more than one litre of pleural fluid) are recommended to confirm the causation between pleural effusion and dyspnoea; pleural manometry can be used to assess the mechanical characteristics of the lungs and determine the probability of lung re-expansion after the procedure. In asymptomatic patients, the need for pleural fluid drainage is still debated; according to the 2018 ATS/STS/STR clinical practice guideline, invasive pleural interventions for therapeutic purposes only; however, are not recommended in asymptomatic patients with malignant pleural effusion [2]. Efforts have been made to identify biomarkers for the early diagnosis of mesothelioma. High levels of soluble mesothelin have been associated with MPM (particularly with tumour dimensions and disease progression in the epithelioid subtype), but it is more significant when measured on pleural fluid than on serum, limiting its importance and its clinical application as an early diagnostic biomarker [11]. Other markers, such as plasmatic fibulin-3, have been studied but require further validation before they are included in the common clinical practice.

The analysis of pleural fluid may provide data suggesting the diagnosis of mesothelioma, but has a quite poor sensitivity (around 30%, down to 16% in some studies). Cytology remains, however, the least invasive and most used first-line diagnostic test in case of pleural effusion and suspected malignancy. The diagnostic accuracy of cytological analysis is increased in case of visceral pleural invasion in epithelioid mesothelioma. Immunocytochemical analysis, which can be performed on cell-block sections when a large amount of pleural fluid is sent for evaluation, is often required in order to obtain a diagnosis [12]. It has been demonstrated that the diagnostic sensitivity is improved when submitting a larger quantity of pleural fluid up to 75 mL when performing direct smear, up to 150 mL when using cell-block sections too [13]. Anyway, in case of suspected mesothelioma, despite the common use of cytology for diagnostic purposes, the International Mesothelioma Interest Group strongly suggest the use of a histological sample for the definitive diagnosis [14]. There are several possible procedures which can provide adequate samples for a histological characterization of the disease. Blind closed pleural biopsy is performed using an Abrams or Cope needle; without the aid of a real-time imaging technique, however, the diagnostic accuracy for mesothelioma is unsatisfactory. US or CT-guided biopsy yields a significantly higher sensitivity, but this technique is not recommended as a first-line investigation by the latest ERS/ESTS/EACTS/ESTRO guidelines; however, this image-guided technique can be extremely useful in patients with no pleural effusion [7].

A valid alternative that makes it possible to biopsy pleural irregularities under direct vision is pleuroscopy, also known as medical thoracoscopy: it can be performed with local anaesthesia and under mild sedation and has a low rate of complications. However, minimally invasive surgery, such as video-assisted thoracic surgery (VATS) and thoracoscopy, is the preferred approach in most centres, and particularly by thoracic surgical centres. In fact, this technique it enables to perform multiple biopsies, to better assess the involvement of the different pleural surfaces (parietal, mediastinal, diaphragmatic and visceral) and of the mediastinal region, and to perform therapeutic procedures (pleurodesis, pleurectomy, etc.) during the same operation. It is recommended to perform multiple and deep biopsies of both pleural abnormalities and macroscopically normal tissue, in order to reduce the risk of false negatives and obtain samples of fat and muscle tissue [15,16]. The procedure is usually carried out under general anaesthesia, but non-intubated VATS surgery (NITS) has been reported.

An interesting issue is the high potential of patient-derived xenografts (PDXs) in testing new therapies against MPM, as reported by Affatato et al. [17].

## 5. Treatment

Management of recurrent pleural effusion in malignant pleural mesothelioma still remains a challenge for clinicians, especially when patients suffer from progressive breathlessness and impaired quality of life. The best clinical approach should take into account the extension of the disease, the performance status and the expected survival of the patient, and it should aim to relieve symptoms, reduce hospitalization and improve quality of life with minimally invasive procedures.

Various medical and surgical procedures are available to reduce pleural fluid recurrence and to relieve symptoms: in particular, these include both mechanical and chemical pleurodesis with sclerosing agents, indwelling pleural catheter placement (IPC). If the patient can tolerate a surgical procedure, pleural biopsies and chemical or mechanical pleurodesis may be performed during the same thoracoscopic intervention, after a frozen section diagnosis or confirmation of pleural malignancy.

Chemical pleurodesis is a procedure intended to create a symphysis between the parietal and visceral pleura by intrapleural administration of various chemical agents, such as talc, doxycycline, tetracycline and bleomycin, with a consequent inflammatory response between visceral and parietal pleura that leads to the obliteration of pleural space and prevents effusion recurrence. Among various available sclerosing materials, sterile talc is the most common and the most successful chemical agent for performing pleurodesis. It can be applied under direct vision during thoracoscopy or may be instilled through the pleural drainage (talc slurry); while the former technique can be used only during a surgical procedure, the latter can be administered in suspension form at the bedside through the chest tube and can be also used in patients who are unfit for surgery. In the case of talc slurry, recent studies suggest that higher pleurodesis success rates may be obtained using large (24 Ch) compared to small-calibre chest tubes [18]. VATS pleural biopsies and pleurodesis can be performed also without endotracheal double-lumen intubation in a NITS procedure: this minimally invasive technique can be performed in fragile and high-risk patients, too, with reduced intubation-related risks and a faster recovery [19].

Fysh et al. published a retrospective analysis of 390 patients comparing the success rate of surgical and bedside chemical pleurodesis in MPM pleural effusion, reporting no significant differences in efficacy, patient survival, and total time spent in hospital after the procedure, even though patients who underwent surgical pleurodesis were significantly younger than the bedside group [20]. Pleurodesis was effective only in about 30% of the treated patients in both groups, confirming the lower success rate of pleurodesis in MPM compared to pleural effusion due to other malignancies: this phenomenon could be explained by the fact that MPM usually invades large parts of the pleural surface, altering the physiological drainage of pleural effusion. In addition, pleurodesis efficacy is inversely proportional to life expectancy in MPE: given the fact that MPM is associated with longer survival than other metastatic malignancies causing pleural effusion, pleurodesis can be less effective over the long term [20].

Other described sclerosing agents are some antibiotics such as bleomycin, tetracycline and doxycycline, and Corynebacterium parvum, an inactivated anaerobic gram-positive bacterium associated with an intense immunostimulation.

Mechanical pleurodesis is a surgical procedure aimed at obtaining pleural symphysis by creating abrasions on the parietal pleura and causing an inflammatory process and a scar between parietal and visceral pleura. It is less used than chemical pleurodesis for recurrent malignant pleural effusion.

General contraindications to pleurodesis are conditions in which there is not enough contact between the visceral and parietal pleura, and therefore pleural space cannot be obliterated: in MPM this condition is commonly found in case of non-expandable lung. In these cases, the visceral pleural disease associated with recurrent effusion traps the lung, thus preventing its complete expansion after fluid removal.

Non-expandable lung is common in MPM: a prospective observational cohort study by Bibby et al. including 192 MPM patients with pleural effusion described 64 cases of non-expandable lung (33.3%), associated with significant symptomatology, shorter survival, and a hazard ratio for mortality of 1.80, compared with MPM patients without trapped lung [21].

In these clinical conditions, in which both chemical and mechanical pleurodesis are ineffective, an alternative treatment option can be represented by indwelling pleural catheter placement. Indwelling pleural catheters are small silicone flexible tubes with calibres around 15 Fr, in which the distal part (usually multifenestrated) is inserted in the pleural space, while the medial portion is designed to be located in a subcutaneous tissue tunnel and to create a fibrous reaction that allows the long-term maintenance of the device; the proximal end is connected to a one-way valve through which the patient can periodically (in most cases daily) drain the pleural fluid without any medical supervision. Indications for IPC placement are recurrent malignant pleural effusions in which pleurodesis procedures have been ineffective, and cases on non-expandable lung; IPC should also be considered as a palliative solution when recurrent effusion causes a serious deterioration of clinical conditions and quality of life. IPC also can be placed under local anaesthesia at the bedside or in an outpatient setting: the main advantages are the easy management of the device in out-of-hospital scenarios too, the reduction of hospital stays and readmissions, and the significative improvement in symptoms and patients’ quality of life [22]. General contraindications include pleural or systemic infections potentially leading to sepsis, coagulation disorders, and severe immunosuppression. IPC can be used safely also in patients undergoing chemotherapy: a recent retrospective analysis including 104 IPC patients reported no significant differences between chemotherapy and control group in terms of pleural infections and chest pain [23]. Even if serious complications (mainly including displacement and bleeding) are extremely rare, some possible adverse effects of IPC may occur, namely pleural or subcutaneous tract infections commonly caused by S. aureus requiring antibiotic therapy and often hospitalization. Another described adverse effect is catheter tract metastasis (CTM): a retrospective review by Thomas et al. reported 11 cases of CTM in 107 IPC patients (10% of incidence), occurring mainly in the case of mesothelioma [24]; on the other hand, a systematic review evaluating efficacy and safety of IPC in MPE reported a significantly lower rate of CTM (0.8%) [25]. CTM is discretely responsive to radiotherapy (6 of the 11 above reported cases of CMT were treated with radiation therapy with no significant complications); however, the role of prophylactic catheter tract irradiation in mesothelioma pleural effusion still remains controversial, and is not currently justified [26]. IPC daily drainage may also be effective for obtaining pleural symphysis, keeping the pleural space dry and allowing the contact between visceral and parietal pleura. The timing of IPC drainage is still on debate: a recent multicentre randomized controlled trial including 87 patients from 11 medical centres compared “aggressive” (daily) versus “symptom-guided” approach to IPC drainage in patients with MPE in terms of efficacy in breathlessness control, induction of pleurodesis, quality of life, hospitalization, and complication rates. The obtained results showed no significative differences between the two groups in terms of mortality, breathlessness and pain score, hospital stay and readmission, and adverse events, but significantly more patients in the aggressive arm developed spontaneous pleurodesis (37.2%) and showed a better quality of life [27].

VATS partial pleurectomy (VAT-PP), a surgical manoeuvre of debulking performing parietal pleurectomy with or without decortication can be proposed in selected cases, but some evidence show that VAT-PP does not improve overall survival if compared with talc pleurodesis. In particular, a multicentre RCT comparing VAT-PP and talc pleurodesis in patients with MPE due to MPM reported higher complication rates (respiratory complications, air leak) and a longer hospital stay in patients treated with VAT-PP, with no survival advantage in this group of patients. However, VAT-PP surviving patients at 6 and 12 months showed a better EQ-5D-assessed quality of life score, suggesting that this option might have a role in patients with at least 6 months life expectancy [28].

Recently, new medical therapeutic options are being proposed not only for palliation of MPE, but also to treat the underlying causes of effusion recurrence: in particular VEGF has been recognized as an important factor promoting cancer and mesothelial cells production of secretions and playing a relevant role in the process of MPE formation. In this setting, Chan et al. proposed a novel therapeutic option combining systemic chemotherapy (pemetrexed and cisplatin) with intrapleural injection of pemetrexed and bevacizumab (anti-VEGF antibody). In the 23 patients enrolled, the combined systemic and local chemotherapy effectively reduced effusion, thus relieving symptoms and improving quality of life [29]. Few adverse events related to bevacizumab were registered, mainly bleeding and high arterial blood pressure. Even though further studies are needed to evaluate the real clinical value of intrapleural anti-VEGF therapy, it can be a promising alternative treatment option to reduce recurrent effusion in selected patients.

## 6. Discussion

**Figure 1:**
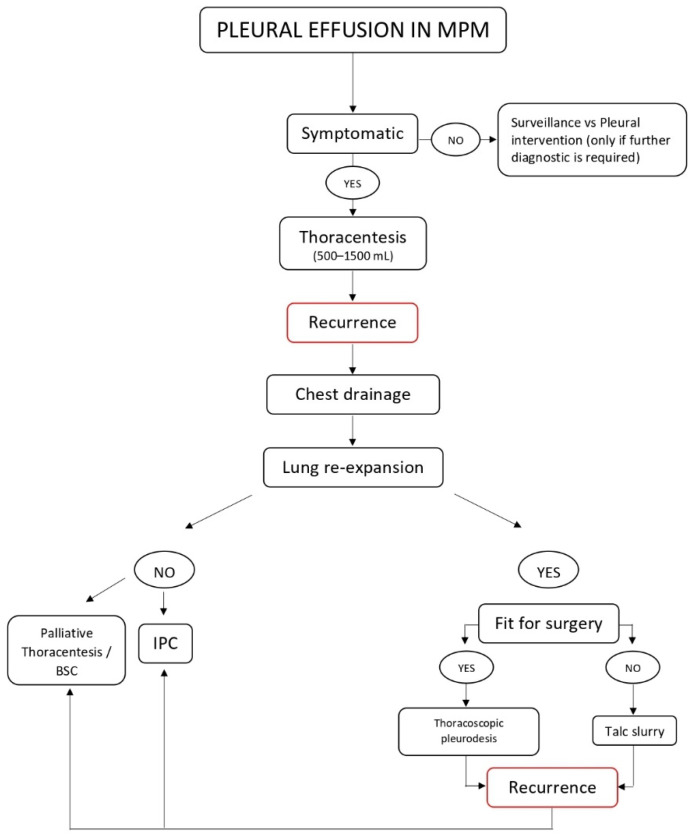
Management of pleural effusion in MPM.

Pleural effusion in MPM often represents the first clinical presentation of the disease, is associated with symptoms such as dyspnoea, progressive breathlessness, worsening of general conditions and quality of life, and still remains a clinical challenge in terms of both therapeutic approach and long-term management. An algorithm for the management of pleural effusion in MPM is depicted in Figure 1.

Various conditions should be taken into account in order to offer the most effective and patient-centred therapeutic option: several surgical procedures may be performed to reduce effusion recurrence and relieve symptoms; thoracentesis and chest drainage are the first-line diagnostic and therapeutic options recommended by international guidelines, but it should be considered that cytologic evaluation alone has a poor diagnostic value especially for MPM [13].

In case of recurrence, a definitive pleural intervention is advised: among the above mentioned procedures, talc pleurodesis is the standard treatment if patient’s life expectancy is higher than 3 months [30]: talc can be administered either during thoracoscopic interventions or through chest drainage at bedside (talc slurry) if the patient cannot tolerate a surgical procedure A recent multicentre randomized clinical trial including 330 patients and comparing the efficacy thoracoscopic talc poudrage compared with talc slurry to manage MPE showed no significant differences in pleurodesis failure at 90 and 180 days, proving that talc slurry can be a valid alternative to thoracoscopy in frail patients who cannot tolerate a surgical procedure [31]. However, pleurodesis has a low success rate in MPM (around 30%), and failure rate increases with longer survival; more than 30% of patients required further pleural interventions after pleurodesis. In such cases talc pleurodesis can be repeated via chest tube, or an indwelling pleural catheter may be inserted. Talc can also be administered through IPC in an outpatient setting: a randomized trial by Bhatnagar et al. including 154 patients (78 talc vs. 76 placebo) reported significantly higher chances of pleurodesis than with IPC alone, without differences in adverse effects [32]. Several studies comparing talc pleurodesis and IPC placement in terms of efficacy in MPE management were published: in particular, a recent meta-analysis by Yeung et al. including four randomized controlled trials reported no significant differences in pleurodesis success rate, dyspnoea improvement, number of adverse events and mortality between the two procedures, with a significative lower hospitalization time for IPC group (weight mean difference: 2.19) [22].

Regarding quality of life, data between the selected studies cannot be compared: nevertheless, two studies reported no significant differences and one reported better quality of life scores in the pleurodesis group, underlying how the question is still under debate and how many factors can affect this hardly measurable endpoint.

## 7. Conclusions

Recurrent pleural effusion in malignant pleural mesothelioma is a clinical challenge in terms of diagnostic approach and long-term management: even though international guidelines have an essential role in clinical decision-making, the best therapeutic option should be patient-tailored, considering also quality of life and time spent in hospitals, given the poor prognosis and life expectancy of these patients, who are often severely symptomatic. In this scenario, we proposed our point of view and our diagnostic-therapeutic approach to MPE in MPM, which can be a helpful practical tool for clinicians dealing with this type of patients. Further research is needed to completely understand MPE pathogenesis, to apply in everyday clinical practice new promising diagnostic and prognostic biomarkers and to provide the best therapeutic or palliative surgical and medical options for each patient.
